# US line-ups outperform UK line-ups

**DOI:** 10.1098/rsos.160300

**Published:** 2016-09-07

**Authors:** Travis M. Seale-Carlisle, Laura Mickes

**Affiliations:** 1 Department of Psychology, Royal Holloway, University of London, Egham, UK; 2 Department of Psychology, University of California, San Diego, CA, USA

**Keywords:** eyewitness identification, US line-up, UK line-up, simultaneous line-up, sequential line-up

## Abstract

In the USA and the UK, many thousands of police suspects are identified by eyewitnesses every year. Unfortunately, many of those suspects are innocent, which becomes evident when they are exonerated by DNA testing, often after having been imprisoned for years. It is, therefore, imperative to use identification procedures that best enable eyewitnesses to discriminate innocent from guilty suspects. Although police investigators in both countries often administer line-up procedures, the details of how line-ups are presented are quite different and an important direct comparison has yet to be conducted. We investigated whether these two line-up procedures differ in terms of (i) discriminability (using receiver operating characteristic analysis) and (ii) reliability (using confidence–accuracy characteristic analysis). A total of 2249 participants watched a video of a crime and were later tested using either a six-person simultaneous photo line-up procedure (USA) or a nine-person sequential video line-up procedure (UK). US line-up procedure yielded significantly higher discriminability and significantly higher reliability. The results do not pinpoint the reason for the observed difference between the two procedures, but they do suggest that there is much room for improvement with the UK line-up.

## 1. Introduction

The USA and the UK are like-minded nations with similarities that extend well beyond their common language. An institutional similarity is in legal systems (e.g. both nations operate under common law) and a cultural similarity is in crime rates [1]. Yet another similarity is that police investigators in both nations often administer a line-up procedure to an eyewitness during the course of a criminal investigation. However, the details of how line-ups are presented in the two nations are quite different, and our goal was to determine if the diagnostic accuracy of the US line-up procedure differs from that of the UK line-up procedure.

A line-up consists of the police suspect, who is either innocent or guilty, and several other individuals, or fillers, who resemble the suspect and are known innocents. Although the US and UK line-up procedures share those general characteristics, they differ in several respects, including the number of line-up members presented (typically six in the USA versus nine in the UK), the presentation of the line-up members’ images (typically photographs in the USA versus video presentations in the UK), and the procedure used to present the line-up (simultaneous or sequential presentation of line-up members). In the USA, the line-up procedure varies among each jurisdiction, but the most common procedure involves the simultaneous presentation of six static photographs, with each photograph showing a front view of a face. In the UK line-up procedure (standardized across England and Wales), videos of nine line-up members are sequentially presented, and each video shows an individual facing forward, and then turning to each side for profile views. Witnesses watch two rounds of each video before making a decision [2].

One difference from the current study and current practices is that statements of confidence are not routinely taken in the UK [2], but are often taken in the USA [3]. We collected confidence (in accordance with a recommendation of the recent National Academy of Sciences committee on the state of eyewitness identification research [4]) and made use of that information to determine which procedure yields higher diagnostic accuracy.

In the USA, sequential line-ups were long thought to be superior in terms of discriminability (the ability of eyewitnesses to distinguish between innocent and guilty suspects) to simultaneous line-ups because they often yield a lower false ID rate, a marginally lower correct ID rate and critically, a higher diagnosticity ratio (correct ID rate/false ID rate) [5–7]. The sequential superiority claim has resulted in up to 30% of US law enforcement agencies changing from the simultaneous to the sequential line-up [3]. Recently, however, it came to light that receiver operating characteristic (ROC) analysis is a more appropriate strategy when the goal is to measure discriminability [4,8–12], but see [13]. The fact that the diagnosticity ratio, a likelihood ratio, does not purely measure discriminability is fairly new to the field of eyewitness identification research, but has been known for decades in other applied fields, such as in diagnostic medicine [14]. When ROC analysis is used, the simultaneous line-up has often been found to outperform the sequential line-up [15–18].

Results of ROC analysis are important for policymakers deciding which type of line-up to use [15]. However, once a criminal case reaches a court of law, regardless of the procedure that was used during the investigation, and regardless of whether one procedure is shown to have greater discriminability than the other, judges and jurors need to know if identifications during the initial line-up procedure are reliable. That is, they need to know the positive predictive value (PPV) of a suspect ID made with a particular level of confidence. ROC analysis does not provide that answer, but an analysis of the confidence–accuracy relationship does.

To measure this relationship, data are typically analysed using calibration analysis or confidence–accuracy characteristic (CAC) analysis. There is consistently a strong confidence–accuracy relationship for individuals who make an identification from a line-up in the laboratory [19–22] and in the field [23,24]. Calibration analyses often involve plotting accuracy for those who identify suspects or fillers [25], but CAC analysis most directly supplies the answer to the question that judges and juries have about a testifying eyewitness who has identified a suspect: how accurate is that suspect identification likely to be given the level of confidence that was expressed?

Two experiments were conducted to compare discriminability and reliability of US and UK line-ups, and they differed only slightly with regard to the UK line-up condition. In one of the experiments (but not the other), after lapping through the line-up twice, participants in the UK condition had the opportunity to view as many line-up members as often as desired before making their decision [2]. Because there were no important differences in the results, we combined the data and present them together (and present the frequency counts separately in table 1). We report the results of both ROC analysis, which evaluates the level of discriminability supported by the US and UK line-up procedures, and CAC analysis, which measures the confidence–accuracy relationship associated with suspect IDs for the two procedures.

**Table 1.** Frequency counts of suspect IDs, filler IDs, no IDs for target-present and target-absent line-ups for every level of confidence for Experiment 1a and 1b. *ID*,*identification*; *SIDs*,*suspect* IDs; *FIDs*,*filler* IDs.

	US condition					UK condition				
	target-present			target-absent		target-present			target-absent	
confidence	SIDs	FIDs	no IDs	FIDs	no IDs	SIDs	FIDs	no IDs	FIDs	no IDs
Experiment 1a										
0	0	4	1	5	6	0	3	10	7	7
10	1	0	0	4	0	0	3	3	1	1
20	2	3	4	4	4	1	5	2	5	2
30	7	10	6	12	6	1	17	4	6	2
40	10	9	5	14	8	3	15	3	12	5
50	15	13	7	25	11	3	19	12	18	8
60	10	12	8	28	11	5	19	6	17	8
70	17	12	18	27	18	11	27	12	21	10
80	17	12	12	12	13	5	13	7	20	10
90	12	5	12	5	19	4	5	10	11	6
100	6	0	5	2	14	7	4	8	9	5
Experiment 1b										
0	1	3	1	6	2	3	7	6	8	4
10	1	3	3	4	0	0	2	2	1	1
20	5	6	4	4	1	1	5	2	7	1
30	5	10	6	13	12	2	8	10	21	4
40	13	7	5	27	11	5	13	4	14	1
50	12	26	16	26	19	6	19	12	33	8
60	21	11	14	22	14	9	20	8	31	11
70	28	14	18	38	31	16	26	17	44	13
80	16	10	13	19	29	13	28	15	36	19
90	13	4	11	12	16	8	14	10	22	11
100	8	0	8	6	17	7	7	2	9	13

## 2. Material and methods

### 2.1. Participants

Participants, undergraduate students from the University of California, San Diego, completed the experiment in exchange for course credit (*N*=2249; 1551 female, 681 male and 17 did not state; age in years: *M*=20.62; s.d.=2.80; ethnicity: Asian 56%, White 19%, Hispanic 15%, Black 1%, other 6% and did not state 2%). Participants were randomly assigned to the US line-up or UK line-up condition, and to a target-present line-up or a target-absent line-up. We determined that a sample size of 1000 (for both Experiment 1a and 1b) would yield sufficient power to detect an effect size as large as the one observed in previous research for simultaneous versus sequential line-ups [17]. Data collection continued until the term ended.

### 2.2. Materials

#### 2.2.1. Video

In a 20 s video of a mock crime, a young White male stole several items from a vacated office. The front of the offender’s face was clearly shown for 8 s.

#### 2.2.2. Line-up construction

An experienced London Metropolitan Police Officer with specialized training in eyewitness identification procedures filmed the actor according to Police and Criminal Evidence (PACE) code specifications [2]. The officer also selected nine fillers based on PACE code guidelines from the PROMAT database (the database used by the London Metropolitan Police Force for constructing line-ups). No specific filler was designated as the innocent suspect in target-absent line-ups. For the US line-up, five or six of the nine fillers were randomly selected for target-present or target-absent line-ups, respectively. For the UK line-up, eight of the fillers were randomly selected for target-present line-ups and all of the nine fillers were used in target-absent line-ups. Positions of the line-up members were randomly set for each participant. The same stimuli were used for both the UK and US line-up procedures.

### 2.3. Procedure

The experiment took place online. After digitally consenting, participants entered demographic information (age, ethnicity, education level), watched the video, played a 5-min game of Tetris as a distractor task, and then were tested on a line-up. They chose someone, or rejected the line-up, from a US or UK line-up (that was target-absent or target-present), and rated their confidence on a 100-point scale (0=*just* guessing and 100=*absolutely* certain). They then answered several multiple-choice questions about the video, including a validation question (What crime was committed?) and were debriefed.

Because the experiment took place online there was no administrator influence. The experiment was programmed so that reloading, pausing and returning to a previous page was disabled; and participants were prevented from participating more than once.

#### 2.3.1. US line-up presentation

In the US line-up condition, photographs of the front view of six line-up members (that were still images of the videos) were presented in a 3×2 matrix. The target’s and fillers’ positions were randomly determined for each participant.

#### 2.3.2. UK line-up presentation

In the UK line-up condition, videos of nine line-up members were presented in sequential order that lapped through twice. The order for both laps was the same for each participant, but the target’s and fillers’ positions were randomly determined for each participant. The line-up took approximately 6 min (depending on Internet connection speed) to complete.

## 3. Results

Participants who incorrectly answered the validation question were excluded from all analyses (*n*=44). Of the 2205 remaining, 571 were in the US target-present condition, 554 were in the UK target-present condition, 577 were in the US target-absent condition and 503 were in the UK target-absent condition. Response frequencies for every level of confidence are in table 1.

### (a) ROC analysis

The overall correct ID rate is the number of suspect IDs from target-present line-ups divided by the number of target-present line-ups presented. The overall false ID rate is the number of estimated suspect IDs from target-absent line-ups divided by the number of target-absent line-ups presented. Because there is no actual innocent suspect in a laboratory study, there are several different approaches to computing the false ID rate. First, one filler in a target-absent line-up can be randomly designated to serve as the innocent suspect (such that any ID of that suspect would count as an innocent suspect ID). Second, the filler who is most often misidentified can be designated as the innocent suspect. A third, now standard, practice [26] estimates the number of innocent suspect IDs from the number of filler IDs from target-absent line-ups. This estimate is obtained by dividing the number of filler IDs by the number of line-up members (six for the US line-up and nine for the UK line-up). That estimated value is then divided by the number of target-absent line-ups to estimate the false ID rate. All three approaches yielded the same conclusions in our study, so we report the results using the third method.

**Figure 1.**
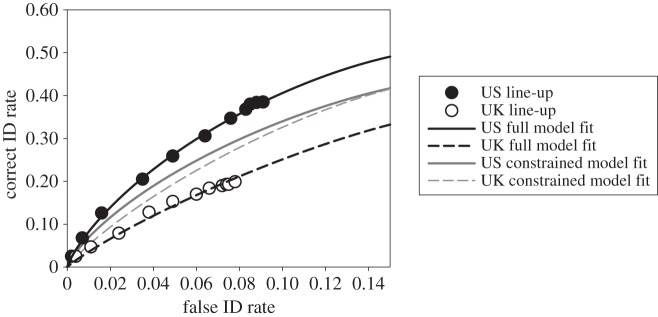
ROC data and curve fits for the US and UK line-up conditions. The solid line represents the fit of the signal detection model and the solid grey line represents the fit of the signal detection model to the US line-up data when *d*′ was constrained to be equal for both conditions. The dashed black line represents the fit of the full signal detection model and the dashed grey line represents the fit of the signal detection model to the UK line-up data when *d*′ was constrained to be equal for both conditions.

The suspect ID rates for target-present line-ups (i.e. correct ID rates), suspect ID rates for target-absent line-ups (i.e. false ID rates) and filler ID rates for both target-present and target-absent line-ups are shown in table 2. The italicized values were used to construct the ROC curves in figure 1. The bold italicized values are the overall correct and false ID rates that have been traditionally analysed in an effort to determine line-up superiority. However, because both the correct ID rate and the false ID rate are lower for the UK procedure (and could, therefore, mean a shift in responding, not a difference in discriminability), an analysis of the full ROC provides a clearer picture of the discriminability associated with the two procedures.

**Table 2.** Suspect IDs, filler IDs and no IDs for target-present and target-absent line-ups rates by level of confidence per condition. *ID*,*identification*; *SIDs*,*suspect* IDs; *FIDs*,*filler* IDs.

		US condition			UK condition		
	confidence	SIDs	FIDs	no IDs	SIDs	FIDs	no IDs
target-present	0	***0.39***	0.30		***0.20***	0.50	
	10	*0.38*	0.29		*0.19*	0.49	
	20	*0.38*	0.29		*0.19*	0.48	
	30	*0.37*	0.27		*0.19*	0.46	
	40	*0.35*	0.24		*0.18*	0.41	
	50	*0.31*	0.21	0.31	*0.17*	0.36	0.30
	60	*0.26*	0.14		*0.15*	0.29	
	70	*0.20*	0.10		*0.13*	0.22	
	80	*0.13*	0.05		*0.08*	0.13	
	90	*0.07*	0.02		*0.05*	0.05	
	100	*0.02*	0.00		*0.03*	0.02	
target-absent	0	***0.09***	0.45		***0.08***	0.62	
	10	*0.09*	0.44		*0.07*	0.60	
	20	*0.09*	0.43		*0.07*	0.59	
	30	*0.08*	0.42		*0.07*	0.57	
	40	*0.08*	0.38		*0.07*	0.52	
	50	*0.06*	0.32	0.45	*0.06*	0.48	0.30
	60	*0.05*	0.25		*0.05*	0.39	
	70	*0.03*	0.17		*0.04*	0.30	
	80	*0.02*	0.08		*0.02*	0.19	
	90	*0.01*	0.04		*0.01*	0.09	
	100	*0.00*	0.01		*0.00*	0.03

Figure 1 shows the ROC curves for both US and UK conditions, and it is apparent that those in the US line-up condition discriminated innocent from guilty suspects better than those in the UK line-up condition. Partial areas under the curve (pAUC) values were computed using a false ID cut-off of 0.078 (the rightmost point for the UK line-up) with the statistical package pROC [27]. The pAUC for US line-up condition (0.017) was significantly greater than the pAUC for UK line-up condition (0.010), *D*=2.74, *p*=0.006. Note that using the rightmost point for the US line-up did not change the conclusion (*p*=0.002).

### 3.2. Model fits

It was recently argued that the results of ROC analysis based on an atheoretical measure like pAUC need not agree with results based on a theoretical measure like *d*′ obtained by fitting a theoretical model to the same data [28]. Although it is theoretically possible for the two approaches to yield different conclusions about which procedure is diagnostically superior, in practice, this is likely to rarely occur. Here, we fitted a theoretical model to the US and UK ROC data and find that, as expected, the atheoretical pAUC analysis and theoretical signal detection analysis agree on which procedure yields higher discriminability.

An equal variance signal detection model was fitted to the data. In the model, memory strength values for innocent suspects (and fillers) and guilty suspects are distributed in two Gaussian, lure and target distributions, respectively, along a memory strength axis. The lure distribution is set to *μ*_*lure*_=0, *σ*_*lure*_=1, and the corresponding mean for the target distribution (*μ*_*target*_, which is the same as *d*′ for the equal-variance model we used) was estimated by fitting the model to the ROC data. A fair target-absent line-up is conceptualized as six or nine random draws (for US or UK target-absent line-ups, respectively) from the lure distribution. A target-present line-up is conceptualized as five or eight random draws (for US or UK target-present line-ups, respectively) from the lure distribution and one random draw from the target distribution (for US and UK target-present line-ups).

To keep the number of parameters down, the observed correct and false IDs were binned into low (ratings from 0 to 60), medium (ratings from 70 to 80) and high (ratings from 90 to 100) confidence levels and were treated as different decision criteria. The full-model estimates values of *d*′, variance (fixed to 1 for both lures and targets), and low, medium and high levels of criteria (c1, c2 and c3, respectively). We also found it necessary to include another parameter *δ*, such that c1, c2 and c3 represent the confidence criteria for target-absent line-ups, and those values divided by *δ* represent the confidence criteria for target-present line-ups. Allowing the criteria to differ in this way is mathematically equivalent to keeping them fixed and instead allowing the standard deviation of the filler distribution to differ for target-present and target-absent line-ups (perhaps because fillers are processed differently when there is a familiar target in the line-up). No conclusions would change if this parameter was omitted from the analysis, but the overall fits would be worse.

The eight parameters were adjusted until the difference between the observed and predicted values was minimized using a *χ*^2^ goodness-of-fit statistic. The fit was very good: the minimum *χ*^2^ goodness-of-fit statistic was not significant, *χ*^2^(8)=4.93, *p*=0.765. Figure 1 shows the observed points from the data and the predicted curves generated from the full signal detection model from both conditions and, for comparison, also shows the curves generated from the signal detection model with the *d*′ parameter constrained to be equal in both conditions. This resulted in a much poorer fit, *χ*^2^(7)=31.30, *p*=0.001, that is significantly worse than when the *d*′ parameter is free to vary, *p*<0.001. The fact that the fit is significantly worse when *d*′ is constrained means that *d*′ for the US and UK procedures differ significantly (in agreement with the results from pAUC analysis).

### 3.3. Comparing discriminability of repeated viewings

We measured whether discriminability for those participants who opted to view line-up members again (*n*=128) differed from those who did not. To do so, we computed *d*′ from the overall correct and false ID rates and compared them using the *G* statistic. We used this approach instead of ROC analysis because separating the data in this manner resulted in too few observations to perform a meaningful pAUC analysis [29]. Those who viewed line-up members more than the required two times had lower discriminability (*d*′=0.35) than those who viewed the line-up members twice (*d*′=0.68), but the difference was not significant, *G*=1.14, *p*=0.253.

### 3.4. Analysis of the confidence–accuracy relationship

**Figure 2.**
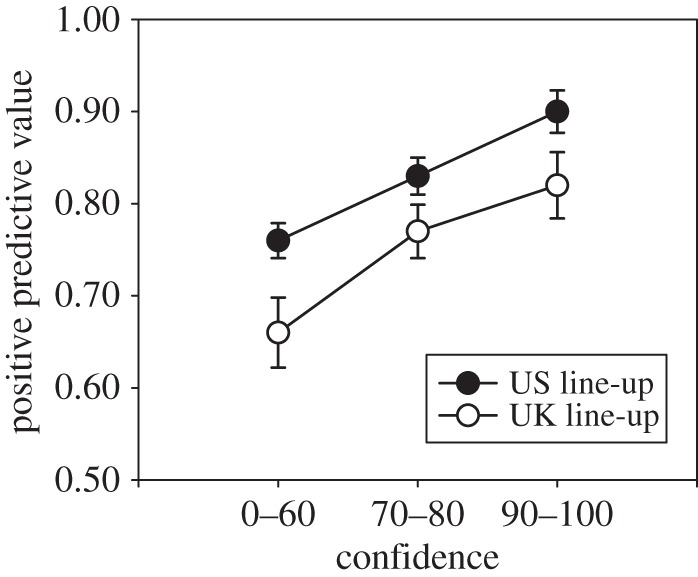
CAC plots for the US and UK line-up conditions. Bars represent standard errors bars estimated using a bootstrap procedure.

Figure 2 shows the CAC curves for the US and UK line-up conditions. The CAC-dependent *variable*=(*number* *of* *correct* *suspect* *IDs*)/(number of correct suspect *IDs*+*number* of incorrect suspect IDs) for every level of confidence, where incorrect suspect IDs refers to the estimated innocent suspect IDs obtained in the manner previously mentioned. Levels of confidence were collapsed into three bins because there were too few responses in certain bins (the same bins that were used for model fitting). The dependent measure is the PPV, that is, it is the probability that a suspect who was identified by a witness truly is the perpetrator. Consistent with recent findings, PPV for both conditions increased with confidence [19]. PPV for the UK line-up condition was lower for each level of confidence than for the US line-up condition. The non-overlapping standard errors in figure 2 indicate that the suspect ID accuracy scores for the US condition were reliably higher than the corresponding values from the UK condition at each level of confidence.

Note that the PPV values in figure 2 correspond to the approximately 50% base rate of target-present line-ups used in this experiment. In the real world, the base rate of target-present line-ups is unknown. The PPV values in figure 2 would be higher for base rates greater than 50% and lower for base rates less than 50%, but the relative standing of the two procedures would not change so long as the base rates were the same for both procedures.

### 3.5. Computing confidence–accuracy characteristic standard errors

The standard errors associated with suspect ID accuracy scores cannot be directly computed and were therefore estimated using a 10 000-trial bootstrap procedure. On each trial, the observed data from target-present (TP) line-ups were randomly sampled with replacement to obtain a bootstrap sample of suspect IDs for that trial. For example, for the observed TP data in the US condition, there were 150 high-confidence suspect IDs out of 500 line-ups, so the observed high-confidence suspect ID hit *rate*=150/500=0.30. Thus, on each bootstrap trial, a high-confidence suspect ID was registered with probability 0.30 for each of 500 line-ups (i.e. a high-confidence suspect ID would be registered approximately every third line-up, on average). The first bootstrap trial might yield 157 suspect IDs, the next bootstrap trial might yield 141 suspect IDs and so on. Similarly, on each bootstrap trial, the observed data from target-absent (TA) line-ups were randomly sampled with replacement to obtain a bootstrap sample of filler IDs for that trial. For example, for the observed TA data in the US condition, there were 100 high-confidence filler IDs out of 500 line-ups, so the observed high-confidence filler ID hit *rate*=100/500=0.20. Thus, on each bootstrap trial, a high-confidence filler ID was registered with probability 0.20 for each of 500 line-ups (i.e. approximately every fifth line-up yielded a high-confidence filler ID). The first bootstrap trial might yield 94 filler IDs, the next bootstrap trial might yield 101 filler IDs and so on. After obtaining a bootstrap sample of suspect IDs and filler IDs on a given bootstrap trial, a suspect ID accuracy score was computed in exactly the same manner it was computed for the observed data. Thus, for example, if there were 157 suspect IDs and 94 filler IDs on the first bootstrap trial, then suspect ID accuracy for the first bootstrap *trial*=157/(157+94/6)=0.909. Note that the bootstrap sample of 94 filler IDs was divided by line-up size (six) to estimate innocent suspect IDs from target-absent line-ups. Similarly, if there were 141 suspect IDs and 101 filler IDs on the second bootstrap trial, then suspect ID accuracy for the second bootstrap *trial*=141/(141+101/6)=0.893. This process was repeated for 10 000 bootstrap trials, and the standard deviation of the 10 000 bootstrap suspect ID scores provided the estimated standard error. The same procedure was followed for each confidence level separately in the US condition and for each confidence level separately in the UK condition.

## 4. Discussion

Line-up procedures used in the USA and UK were not developed by scientists and then implemented in the field, but were instead developed by law enforcement agencies who have no objective basis for preferring one procedure to another. The best way to determine which procedure is diagnostically superior is to use ROC analysis [15]. In the first direct comparison of US and UK line-up procedures using ROC and CAC analysis, we found that the US line-up yielded significantly higher discriminability and significantly higher accuracy at each level of confidence.

The two procedures differ in several ways (e.g. dynamic versus static images, nominal line-up size) so it is not possible to say why the US procedure outperformed the UK procedure. Our findings may be another example of the often-replicated difference between US sequential photo line-ups and US simultaneous photo line-ups, which generally favour the latter. Alternatively, participants in the UK condition (and with sequential line-ups more generally), but not in the US condition (or with simultaneous line-ups more generally), may lose attention during the course of the protracted line-up procedure. The UK line-up takes about 6 min before a decision could be made whereas a decision could be made within seconds after presentation of the US line-up. Could this difference, that is inherent to the procedures, explain the results? Retention interval can be construed as time from end of the video presented during the study phase to the average time the target is first presented during the test phase. For the US procedure, it is approximately 5 min (i.e. the duration of the distractor task). For the UK procedure, it is approximately 5 min in addition to the time to get to position four or five in the line-up (the average position of the target). Each line-up member’s video lasts approximately 15 s, thus, on average, the UK procedure would add about an extra minute to the retention interval. Such a small difference in retention interval is unlikely to account for the differences, nonetheless it is a possibility worth considering.

Whatever the reason for the difference, these findings underscore the importance of directly comparing line-up procedures in terms of their ability to discriminate innocent from guilty suspects. Using a line-up constructed by an experienced police officer for one set of stimuli and one exposure duration, we found that the US procedure unambiguously outperformed the UK procedure. Although it seems unlikely that our results are specific to the testing conditions used here, future work should investigate a wide range of stimuli and conditions (to more definitively answer the applied question of which procedure is superior), and it should also investigate the specific source of the difference between the diagnostic accuracy of the two procedures (to facilitate theory development). Given how many innocent and guilty suspects are tested using line-up procedures in both the USA and the UK, such work should be an urgent priority.

## Data accessibility

The entire dataset is accessible at http://dx.doi.org/10.5061/dryad.1j84f.
